# Health inequity assessment in Brazil: is EQ-5D-3L sensible enough to detect differences among distinct socioeconomic groups?

**DOI:** 10.1186/s12955-024-02235-0

**Published:** 2024-02-27

**Authors:** Bernardo Rangel Tura, Milene Rangel da Costa, Sylvia Lordello, Danillo Barros, Yuri Souza, Marisa da Silva Santos

**Affiliations:** 1 Centre of Health Technology Assessment, National Institute of Cardiology, Rio de Janeiro, Brazil; 2 Faculty of Pharmacy, Federal University of Rio de Janeiro, Rio de Janeiro, Brazil; 3 Instituto Dara, Rio de Janeiro, Brazil

**Keywords:** EQ-5D-3L, EuroQol, Health-related quality of life, Self-reported health, Health inequity, Equity, Sociodemographic indicators

## Abstract

**Background:**

Multidimensional health-related quality of life (HRQOL) instruments, such as the EQ-5D, are increasingly used to assess inequalities in health. However, it is necessary to explore the ability of these instruments to capture differences between population groups, especially in low/middle-income countries. This study aimed to investigate whether the EQ-5D-3L instrument can detect differences in HRQOL between groups of different socioeconomic status (SES) in Brazil.

**Methods:**

Data collection occurred during the Brazilian EQ-5D-3L valuation study and included respondents aged 18 to 64 years enrolled in urban areas. SES was aggregated into three categories: “higher” (A and B), “intermediate” (C) and “lower” (D and E). EQ-5D-3L index was calculated considering the Brazilian value set. A mixed-effects regression model was estimated with random effects on individuals and marginal effects on SES, sex, and educational attainment. Odds ratios for the chance of reporting problems for each EQ-5D dimension were estimated by logistic regression.

**Results:**

A total of 9,148 respondents were included in the study. Mean age was 37.80 ± 13.13 years, 47.4% were men and the majority was ranked as classes B or C (38.4% and 50.7%, respectively). Participants in lower SES classes reported increasingly poorer health compared to individuals in higher classes. The mean EQ-5D-3L index decreased as SES deteriorates being significantly higher for classes A and B (0.874 ± 0.14) compared to class C (0.842 ± 0.15) and classes D and E (0.804 ± 0.17) (*p* < 0.001). The same was observed for the mean EQ-VAS scores (84.0 ± 13.8 in classes A and B, 81.0 ± 17 in class C and 78.3 ± 18.7 in class C [*p* < 0.001]). The multivariate analysis confirmed that SES is an independent factor that effects EQ-5D-3L index measures. Participants in intermediate and lower SES classes have a statistically significant lower EQ-5D-3L index compared to participants in classes A and B, regardless of age, sex, and educational attainment.

**Conclusion:**

In a Brazilian population sample, the EQ-5D-3L instrument was able to detect important differences between groups with distinct socioeconomic statuses (SES). The EQ-5D-3L is useful for exploring inequities in health.

## Background

Health inequity refers to unfair and avoidable differences in health status between population groups. It is a situation in which people are not able to achieve their full health potential due to a lack of access to healthcare and other basic needs, such as healthy food, safe drinking water, housing, and even employment and social support [1] Inequity has a close connection to social characteristics, and the place a person is born, works, and lives impacts their health outcomes, quality of life, and well-being [2].

Social inequalities disproportionately affect vulnerable and marginalized populations that are frequently exposed to risky life and work conditions. These conditions lead to higher morbidity rates [3], especially in the context of public health emergencies [4]. The COVID-19 pandemic is a good example of how health inequity is shaped by political-economic interests. It has been shown that socioeconomic conditions are a major determinant of negative outcomes for COVID-19 patients, regardless of other risk factors such as age or comorbidities [5].

The cornerstone of all universal-coverage countries is to ensure equal access to healthcare [6]. Achieving this goal requires programs that prioritize vulnerable populations and, consequently, promote equity [7]. Understanding and measuring health inequalities is essential to develop and implement efficient public health policies [8].

Health-related quality of life (HRQOL) measures are widely used to inform decision-makers about optimal resource allocation. There are different instruments designed to measure HRQOL. Among them, the EQ-5D is the most frequently used which consists of a standardized questionnaire developed to produce a generic measure of health that could be used in clinical and economic studies about various health conditions [9]. The EQ-5D-3L version was created in 1990 and comprises 2 components: the EQ-5D descriptive system and the EQ visual analogue scale (EQ-VAS). The first includes five dimensions (mobility, self-care, usual activities, pain/discomfort, and anxiety/depression) and for each question in the EQ-5D-3L, there are three response options: no problems, moderate problems, or extreme problems. The responses are converted into a single score or utility index that can be used for different applications, such as the calculation of quality-adjusted life-years (QALYs) in economic evaluations. The EQ-VAS is a numerical scale that measures general health status from the patient’s perspective [9].

More recently, the potential of EQ-5D data to study health inequalities has been explored. For instance, it is possible to investigate how age, sex, and educational attainment impact HRQOL by computing odds ratios based on EQ-5D scores. HRQOL is also applied to the calculation of the concentration index, which is a measure of income-related inequality in health [9, 10].

Equity in health is currently a fundamental principle for health technology assessment, a multidisciplinary process to determine the value of health technologies and inform decision-making to promote efficient health systems [11]. In this context, it is important to explore the ability of instruments designed to measure HRQOL to capture differences between population groups, especially in low-income countries [12]. This study aimed to address this issue by investigating whether the EQ-5D-3L questionnaire can detect the differences in HRQOL between groups of different socioeconomic statuses SES in Brazil. For that, we analyzed whether SES is an independent factor that effects EQ-5D-3L index measures.

## Methods

Data collection occurred from mid 2011 to 2012 during the Brazilian EQ-5D-3L valuation study [13] and the details of this survey and its design have been previously described [13–15]. Respondents aged 18 to 64 years old were interviewed face-to-face. They were enrolled in urban areas of four Brazilian cities (Belo Horizonte, Rio de Janeiro, Porto Alegre, and Recife) according to a geographically based probabilistic sample of the general population from these areas. According to the Brazilian Institute of Geography and Statistics, in 2010, approximately 85% of the Brazilian population lived in urban areas. The states from which data were collected represent four of the seven most populous Brazilian states and account for approximately 30% of the total population. The sample-size definition was based on the desired number of observations for each pair of health states. The sample was selected based on quota sampling by age and sex. The present study included the respondents reporting moderate or extreme problems for any EQ-5D-3L dimensions. The EQ-5D-3L index or utility was calculated according to the questionnaire manual considering the Brazilian value set [14].

A descriptive analysis of respondents’ baseline characteristics was performed computing means and standard deviations (SDs) or medians and interquartile ranges (IQRs). For categorical variables, absolute and relative frequencies were calculated. The proportion of participants reporting problems (moderate or extreme) for each EQ-5D-3L dimension as well as EQ-5D-3L indexes (utilities) and EQ-VAS measures were calculated. Differences were tested at a 5% significance level. For numerical variables ANOVA or Kruskal-Wallis tests were performed. In the case of categorical variables, differences were tested by Chi-square or Fisher’s exact tests.

Socioeconomic status (SES) was defined based on the Brazilian Economic Classification Criteria (Brazilian Criteria) from ABEP—Brazilian Association of Survey Companies (available at https://www.abep.org/criterio-brasil). This classification comprises 6 categories (A, B1, B2, C1, C2, D, and E) defined according to durable assets ownership, householder education attainment, and access to public utility services. Category A corresponds to the wealthiest class, with an estimated average household income of US$4,988 a year. Categories D and E are the most impoverished classes, with an estimated average household income of US$376. For analytical purposes, SES classes were aggregated into three groups referred to as “higher” (A, B1, B2), “intermediate” (C1, C2 categories) and “lower” (D and E categories). Data about SES were gathered from questionnaires that also included SES nonspecific questions about sex, age, religious beliefs, marital status, health insurance, and self-reported health problems.

To investigate if the EQ-5D-3L instrument is sensible enough to detect differences in HRQOL between SES classes in Brazil, we tested the differences of EQ-5D-3L and EQ-VAS scores, as well as the prevalence of problems for each EQ-5D dimension, between socioeconomic groups. Multivariate analysis was performed to test if SES is an independent factor that effects EQ-5D-3L index measures. For that, a mixed-effects regression model was estimated with random effects on individuals and marginal effects on selected variables with EQ-5D indexes as a dependent variable. The selection of the covariates to be included in the model was based on a logistic regression that computed the odds ratios and 95% confidence intervals for the chance of reporting problems for each EQ-5D dimension by different subgroups of participants.

## Results

A total of 9,148 respondents were included in the study. The mean age of the participants was 37.80 ± 13.13 years, and less than half (47.4%) were men. About 50.8% were married, 67.9% had children and 21,2% had college degree. The great majority of the respondents were ranked as classes B or C (38.4% and 50.7%, respectively), 5,1% were classified as class A, 5,6% class D and 0.1% were classified as class E. Sociodemographic characteristics of the respondents are described in Table 1.

**Table 1:** Sociodemographic and clinical characteristics of the participants

Characteristic	Overall N = 9,148	Socioeconomic status			p-value
		A and B N = 3,973 (5.1% and 38.4%)	C N = 4,630 (50,7%)	D and E N = 527 (5,6% and 0.1%)	
*Sociodemographic*					
Sex (N, %)					
Women	4,808 (52.6)	1,966 (49,5)	2,512 (54,4)	310 (58.8)	< 0.001
Men	4,340 (51.6)	2,007 (50.5)	2,118 (53,4)	217 (57,8)	< 0.001
Age (mean ± SD)	37.8 ± 13.1	38.4 ± 13.1	37.0 ± 13.1	40.1 ± 13.5	< 0.001
Marital status (N, %)					
Married	4646 (50.8)	2124 (53.5)	2253 (48.7)	258 (49.0)	< 0.001
Divorced	739 (8.1)	298 ( 7.5)	382 ( 8.3)	57 (10.8)	< 0.001
Single	3497 (38.2)	1470 (37.0)	1840 (39.7)	182 (34.5)	< 0.001
Widow	263 (2.9)	79 ( 2.0)	154 ( 3.3)	30 ( 5.7)	< 0.001
With children	6208 (67.9)	2517 (63.4)	3250 (70.2)	427 (81.0)	< 0.001
College degree (N, %)	1,935 (21.2)	1655 (41.7)	270 ( 5.8)	4 (0.8)	< 0.001
Health insurance (N, %)	3656 (40.0)	2408 (60.6)	1180 (25.5)	59 (11.2)	< 0.001
*Clinical*					
Comorbities (N, %)					
Hypertension	2153 (23.6)	862 (21.7)	1109 (24.0)	175 (33.3)	< 0.001
Lung diseases	2091 (22.9)	1012 (25.5)	980 (21.2)	93 (17.6)	< 0.001
Depression	1048 (18.1)	462 (16.8)	525 (19.0)	57 (23.8)	0.007
Back pain	1606 (17.6)	678 (17.1)	803 (17.4)	119 (22.6)	0.007
Arthritis	678 (7.4)	274 ( 6.9)	334 ( 7.2)	65 (12.3)	< 0.001
Heart diseases	544 (6.0)	227 ( 5.7)	278 ( 6.0)	37 (7.1)	0.467
Diabetes	487 (5.3)	193 ( 4.9)	254 ( 5.5)	36 (6.8)	0.113
Renal failure	170 (1.9)	38 ( 1.0)	106 ( 2.3)	26 (4.9)	< 0.001
Cancer	57 (1.0)	38 ( 1.4)	15 ( 0.5)	2 (0.8)	0.006
Tuberculosis	42 (0.5)	11 ( 0.3)	27 ( 0.6)	4 (0.8)	0.065
HIV	21 (0.4)	10 ( 0.4)	10 ( 0.4)	1 ( 0.4)	0.989
Cirrhosis	18 (0.2)	2 ( 0.1)	15 ( 0.3)	1 (0.2)	0.017
Smoker	3,557 (38.9)	1,361 (34.3)	1,882 (40.6)	305 (57.9)	< 0.001

SD, standard deviation

Self-reported health was considered good or very good by 72.4% of respondents, and only 2% considered themselves to be in poor health (Table 2). The mean EQ-5D-3L index was 0.854 ± 0,15, and the mean EQ-VAS score was 82.1 ± 15.9. The prevalence of problems according to the five dimensions of the EQ-5D was higher for pain/discomfort (45.7%) and anxiety/depression (33.1%). Approximately 11% of the participants reported problems for mobility or usual activities and only 3.3% regarding self-care. The most frequently reported health problem was hypertension (23.6%), followed by lung diseases (22.9%) and depression (18.1%). These results are summarized in Table 2.

**Table 2:** Health-related quality of life measures reported by the participants

Characteristic	Overall N = 9,148	Socioeconomic status			p-value
		A and B N = 3,973 (5.1% and 38.4%)	C N = 4,630 (50,7%)	D and E N = 527 (5,6% and 0.1%)	
*Self-evaluation of general health (N,%)*					
Very good	2032 (22.2)	1106 (27.8)	842 (18.2)	77 (14.6)	< 0.001
Good	4587 (50.2)	2077 (52.3)	2294 (49.6)	211 (40.0)	< 0.001
Regular	2283 (25.0)	725 (18.3)	1340 (29.0)	214 (40.6)	< 0.001
Bad	186 ( 2.0)	53 ( 1.3)	111 ( 2.4)	20 (3.8)	< 0.001
*Proportion of participants reporting problems for EQ-5D dimensions (N,%)*					
mobility	1044 (11.4)	325 ( 8.2)	612 (13.2)	103 (19.5)	< 0.001
Self-care	299 ( 3.3)	78 ( 2.0)	190 ( 4.1)	30 (5.7)	0.002
Usual activities	1031 (11.3)	350 ( 8.8)	594 (12.8)	85 (16.1)	< 0.001
Pain/discomfort	4184 (45.7)	1617 (40.7)	2249 (48.6)	308 (58.4)	< 0.001
anxiety/depression	3032 (33.1)	1237 (31.1)	1577 (34.1)	213 (40.4)	< 0.001
*Health related quality-of life (mean ± SD)*					
EQ-VAS	82.1 ± 15.9	84.0 ± 13.8	81.0 ± 17.0	78.3 ± 18.7	< 0.001
EQ-5D-3L	0.854 ± 0.15	0.874 ± 0.14	0.842 ± 0.15	0,804 ± 0.17	< 0.001

SD, standard deviation

Participants in intermediate (C) and lower (D and E) SES classes reported increasingly poorer health compared to individuals in higher classes (A and B). The prevalence of comorbidities increases with the worsening of SES for almost all reported diseases as well as the proportion of patients reporting problems for each one of EQ-5D-3L dimensions (Table 2). Accordingly, the mean EQ-5D-3L index was significantly higher among participants from classes A and B (0.874 ± 0.14) compared to individuals in classes C (0.842 ± 0.15) and classes D and E (0.804 ± 0.17) (*p* < 0.001). The same was observed for the mean EQ-VAS scores which was equal to 84.0 ± 13.8 in classes A and B, 81.0 ± 17 in class C and 78.3 ± 18.7 in class C (*p* < 0.001).

A logistic regression was performed to identify potential confounders in the relationship between HRQOL and SES. It showed that sex, age, and educational attainment could also significantly affect EQ-5D-3L results (Table 3). Women had higher odds than men of reporting problems in any of the EQ-5D dimensions, especially for pain and depression (OR = 2.14 [95%CI 1.96 to 2.32] and OR = 1.95 [95%CI 1.78 to 2.13], respectively) (Table 3). The odds of reporting mobility or self-care problems were almost 6 times greater for people aged 24 or older. Respondents without a college education reported more problems across all 5 EQ-5D-3L dimensions, mainly for self-care (OR = 3.47 95%CI 2.24 to 5.37]).

**Table 3:** Odds ratios for reporting problems for each of EQ-5D dimensions

EQ-5D Dimension	Variable	Unadjusted OR (95%CI)
Mobility	Men	1.46 (1.28 to 1.66)
	Age < 24 years old	5.95 (4.43 to 7.99)
	College degree	2.13 (1.75 to 2.59)
Self-care	Men	1.57 (1.23 to 1.99)
	Age < 24 years old	5.78 (3.31 to 1.01)
	College degree	3.47 (2.24 to 5.37)
Usual activities	Men	1.37 (1.20 to 1.57)
	Age < 24 years old	3.69 (2.89 to 4.71)
	College degree	1.87 (1.55 to 2.26)
Pain/discomfort	Men	2.14 (1.96 to 2.32)
	Age < 24 years old	1.75 (1.57 to 1.95)
	College degree	1.65 (1.49 to 1.83)
Anxiety/depression	Men	1.95 (1.78 to 2.13)
	Age < 24 years old	1.62 (1.44 to 1.82)
	College degree	1.13 (1.01 to 1.26)

OR, odds ratio; CI, confidence interval

To test whether SES is an independent factor that effects EQ-5D-3L index measures, a multivariate analysis was performed (Table 4). The results confirmed that participants in intermediate and lower SES classes have a statistically significant lower EQ-5D-3L index compared to participants in classes A and B, regardless of age, sex, and educational attainment. This means that EQ-5D-3L instrument was sensible enough to detect differences in HRQOL among individuals with different SES classes.

**Table 4:** Mixed-effects regression model of sociodemographic status, sex, and educational attainment effects on EQ-5D-3L index measure

Coefficient	Coefficient Value	95% confidence interval		SD	p-value
		Lower	Upper		
Intercept	0.93876	0.92818	0.94934	0.00540	< 0.0001
Male	0.04909	0.04329	0.05489	0.00296	< 0.0001
College degree	0.02942	0.02152	0.03731	0.00403	< 0.0001
Age	-0.00264	-0.00286	-0.00242	0.00011	< 0.0001
SES class C	-0.02249	-0.02911	-0.01587	0.00338	< 0.0001
SES classes D and E	-0.04874	-0.06195	-0.03552	0.00674	< 0.0001

SES, socioeconomic status; SD, standard deviation

## Discussion

This is the first study that explored the potential of the EQ-5D-3L to identify health inequalities in Brazil. We found that EQ-5D-3L index and EQ-VAS score were increasingly lower as SES deteriorates. Participants in lower SES classes reported more problems for all EQ-5D dimensions and presented higher rates of chronic conditions, smoking, and lower educational levels. Individuals with lower SES are typically exposed to unhealthy living conditions characterized by the lack of sanitation and clean water, presence of disease vectors [16–19] and impaired access to public health [20, 21] which results in a negative impact in all EQ-5D dimensions [22–24]. These individuals also present a worse perception of their own health as demonstrated in studies that assessed the association between low income or unemployment with HRQOL [25].

Although there is plenty of evidence showing the association between HRQOL and SES, the effect of potential confounders is not usually addressed [26]. This is the case of SES indicators, such as education, that are commonly applied as SES proxies [26]. Spronk et al. explored the usefulness of the EQ-5D-5 in health inequality analyses using the level of education as a proxy for SES and found the worst EQ-5D-5 L sum scores for respondents with lower educational levels [27]. Mielck et al. reached similar conclusions in analyzing the impact of educational level on HRQOL in Germany [28]. Lower education levels were an important predictor of lower EQ-VAS scores even within subgroups of individuals who carry the same chronic disease, meaning that the impact of SES on quality of life goes beyond the prevalence of chronic conditions.

Nevertheless, in our study we used a SES index instead of indicators to capture the most impoverished subgroups of people. Indexes better represent the socioeconomic status since other factors such as nonmonetary income or informal income could also affect the individual socioeconomic level [29]. Importantly, the multivariate analyses showed that SES is an independent factor that affects EQ-5D scores after controlling for other factors such as age, sex, and educational attainment. These findings suggest that the EQ-5D-3L instrument is sensible enough to discriminate groups with different health conditions according to their socioeconomic status in Brazil. Kind et al. [30] also concluded that EQ-5D is capable of detect differences in the health status of different subgroups from a representative sample of the United Kingdom population. They found that the rate of reported problems was up to 120% higher among those from lower social classes, specially for pain/discomfort and anxiety/depression. This subgroup also presented consistently lower EQ-VAS scores regardless of age.

Socioeconomic inequities disproportionately affect vulnerable and marginalized populations, and the COVID-19 pandemic has shed light on the impact of this inequality on health outcomes. The Brazilian study from Santos et al. found a 32% increase in COVID-19 mortality among socially vulnerable individuals compared to those with better SES [3]. In this context, monitoring health inequality is essential for planning public policies that aim to reduce unfair differences for socially disadvantaged groups. And, for that, our results showed that EQ-5D could be a useful instrument.

This study has some limitations. It was based on a domiciliary face-to-face interview, which could have led to the selection of individuals who stay at home during business hours. The SES and health status information were self-reported, and because of that, these data are subjected to classification bias. Also, despite employing a probabilistic sample of four important Brazilian cities, it included mainly individuals from urban areas, which might not be necessarily representative of the Brazilian population. Finally, data collection occurred in 2012. However, valuation studies are time consuming and require a substantial amount of resources to be completed and are not routinely updated.

It is also worth noting that, currently, health equity is an important subject in the field of health technology assessment (HTA), especially in the development of methodological approaches that enable the consideration of health equity into economic evidence to support the public decision-making process [4]. For that, understanding how health-related quality-of-life instruments can capture inequalities is essential. The analysis of the trade-offs between the costs of the interventions and their impact on health for vulnerable subgroups is necessary to support public health initiatives that enhance equity and efficiency in health.

## Conclusion

Considering a Brazilian population sample, the EQ-5D-3L instrument was able to detect important differences between groups with distinct socioeconomic statuses (SES). Lower SES is significantly associated with decreased EQ-5D-3L index and EQ-VAS scores. Therefore, EQ-5D-3L is a useful tool to explore inequities in health and could be applied in health technology assessment to improve health equity, particularly for vulnerable populations.

## Data Availability

The datasets used and/or analyzed during the current study are available from the corresponding author upon reasonable request.

## Abbreviations

ABEP: Brazilian Association of Survey Companies
95%CI: 95% confidence interval
EQ-5D-3L: 3-level version of the standardised measure of health-related quality of life developed by the EuroQol Group
EQ-VAS: EuroQol visual analogue scale
HRQOL: health-related quality of life
OR: odds ratios
QALYs: quality-adjusted life-years
SES: socioeconomic status
SD: standard deviation
